# Clinical Implications of Antiviral Resistance in Influenza

**DOI:** 10.3390/v7092850

**Published:** 2015-09-14

**Authors:** Timothy C. M. Li, Martin C. W. Chan, Nelson Lee

**Affiliations:** 1 Division of Infectious Diseases, Department of Medicine and Therapeutics, Faculty of Medicine, The Chinese University of Hong Kong, 9/F, Clinical Sciences Building, Prince of Wales Hospital, Shatin, Hong Kong, China; liman1986@gmail.com; 2 Department of Microbiology, Faculty of Medicine, The Chinese University of Hong Kong, 1/F, Clinical Sciences Building, Prince of Wales Hospital, Shatin, Hong Kong, China; martin.chan@cuhk.edu.hk

**Keywords:** antiviral resistance, neuraminidase inhibitors, influenza viruses

## Abstract

Influenza is a major cause of severe respiratory infections leading to excessive hospitalizations and deaths globally; annual epidemics, pandemics, and sporadic/endemic avian virus infections occur as a result of rapid, continuous evolution of influenza viruses. Emergence of antiviral resistance is of great clinical and public health concern. Currently available antiviral treatments include four neuraminidase inhibitors (oseltamivir, zanamivir, peramivir, laninamivir), M2-inibitors (amantadine, rimantadine), and a polymerase inhibitor (favipiravir). In this review, we focus on resistance issues related to the use of neuraminidase inhibitors (NAIs). Data on primary resistance, as well as secondary resistance related to NAI exposure will be presented. Their clinical implications, detection, and novel therapeutic options undergoing clinical trials are discussed.

## 1. Introduction

Influenza is a major cause of severe respiratory infections leading to excessive hospitalizations and deaths globally; annual epidemics (e.g., A/H3N2, A/H1N1, B; due to antigenic drift/shift), pandemics (e.g., A/H1N1_pdm09_; due to genetic re-assortment), and sporadic/endemic avian virus infections (e.g., A/H5N1, A/H7N9; adapted for limited human transmission) occur as a result of rapid, continuous evolution of influenza viruses [1]. Cumulative evidence from randomized controlled trials and numerous observational studies indicate that antivirals may improve viral clearance, shorten illness duration, reduce complications, lower death risks, and reduce disease transmission [1,2,3]; as such, emergence of antiviral resistance is of great clinical and public health concern. Currently available antiviral treatments include four neuraminidase inhibitors (oseltamivir, oral; zanamivir, inhalational; peramivir, intravenous/intramuscular; and laninamivir, inhalational), adamantanes (M2-inibitors; amantadine, rimantadine), ribavirin (viral RNA synthesis inhibitor), and a polymerase inhibitor (favipiravir) [1,4]. Peramivir was recently approved for the treatment of influenza by the Food and Drug Association of the United States (FDA); other countries that have approved the use of this neuraminidase inhibitor (NAI) include Japan, Korea and China [5]. Laninamivir and favipiravir are however only available in Japan; the intravenous formulation of zanamivir is under phase III development and only accessible through a compassionate use program in the hospital setting [4]. These potentially available antiviral agents and their approved indications are listed in Table 1. In this review, we focus on resistance issues related to the use of NAIs, as other agents are rarely used because of limited efficacy or availability. Data on primary resistance, as well as secondary resistance related to NAI exposure, which occurs in human seasonal, pandemic and avian influenza virus infections will be presented. Their clinical implications, detection, and novel therapeutic options undergoing clinical trials will be discussed. The reported incidence of NAI resistance and typical mutations associated with reduction in susceptibility in each virus type/subtype are shown in Table 2.

**Table 1:** Potentially available antiviral agents for treatment and prevention of influenza infections.

	Route of Administration	Availability	Indications
**Neuraminidase inhibitors**			
oseltamivir	oral	commercially available	Treatment of influenza A and B age ≥14 days (adults: dose reduction if CrCl <60 ml/min; children: tiered weight-based regimen if <40 kg)
			Chemoprophylaxis age ≥1 year (with half-treatment dose) ^(a)^
zanamivir	inhalational	commercially available	Treatment of influenza A and B age ≥7 years (contraindicated in underlying respiratory diseases, e.g., chronic obstructive airway disease (COPD), asthma) ^(b)^
			Chemoprophylaxis age ≥5 years (with half-treatment dose)
	intravenous	limited (to a compassionate use program)	N.A. (under Phase III clinical trial)
peramivir	intravenous	limited (USA, Japan, Korea, China)	Treatment of influenza A & B age ≥18 years (single dose application) ^(c)^
laninamivir	inhalational	limited (only in Japan)	Treatment of influenza A and B adults and children ^(b)^ (single dose application)
			Chemoprophylaxis age ≥10 years (with half-treatment dose)
**M2-inhibitors**			
amantadine/rimantadine	oral	commercially available	Not recommended due to resistance in nearlyall circulating influenza A (H3N2, H1N1_pdm09_) and B virus strains
**Polymerase inhibitor**			
Favipiravir	oral	limited (only in Japan)	Treatment of “novel or re-emerging” influenza virus infections age ≥18 years (under Phase III clinical trial)

(a) Use of half-dose regimen as chemoprophylaxis is not generally recommended except in special circumstance (e.g., contraindication to vaccines, controlling institutional outbreaks) due to risk of emergence of resistance. Full-dose treatment for at illness onset in exposed individuals can be considered (see text); (b) Due to risk of bronchospasm. The same precaution may apply to laninamivir. Inhalational therapy, because of lack of systemic availability, may result in therapeutic failure in complicated diseases such as pneumonia [1]; (c) Peramivir is approved by the US FDA for the treatment of uncomplicated influenza (same as oseltamivir and zanamivir) as a single-dose application; clinical trials using multiple-dosing regimens among hospitalized patients showed non-inferiority to oseltamivir [5].

**Table 2:** Antiviral resistance in human infections caused by seasonal, pandemic and avian influenza viruses. Typical mutations associated with clinical resistance are shown.

	Neuraminidase Inhibitors		Adamantanes
	**Oseltamivir**	**Zanamivir**	**Amantadine, Rimantadine**
A/H3N2	<3%	rare	>99%
	R292K, E119V ^(a)^		S31N ^(b)^
A/H1N1 (2007-08)	>99%	rare	rare
	H275Y ^(c)^		
A/H1N1_pdm09_	<3%	rare	>99%
	H275Y ^(c)^		S31N
B	rare	rare	100%
	I221V/T ^(d)^		
A/H5N1	rare	rare	variable ^(b)^
	H275Y ^(c)^		
	**Oseltamivir**	**Zanamivir**	**Oseltamivir**
A/H7N9	data limited	rare	>99%
	R294K ^(e)^		

Incidence of resistance as reported in surveillance studies on clinical samples (rare < 1%) (see text). (a) R292K and E119V (N2 numbering) mutations cause resistance to oseltamivir, and reduce susceptibility to zanamivir and peramivir; (b) S31N (M2 numbering) mutation causes resistance to amantadine and rimantadine. Susceptibility among A/H5N1 isolates varies according to geographical area and clade of virus; (c) H275Y (N1 numbering) mutation causes resistance to oseltamivir and cross-resistance to peramivir; zanamivir, and laninamivir susceptibility are not significantly affected; (d) I221V/T (influenza B numbering) causes reduced susceptibility to oseltamivir but not zanamivir; (e) R294K (N9 numbering) mutation causes resistance to oseltamivir and peramivir, and reduces susceptibility to zanamivir and laninamivir. Incidence of this mutation is unclear but likely infrequent.

## 2. Seasonal Influenza Viruses A/H3N2, A/H1N1, B

On-going surveillance data on seasonal influenza virus strains show that resistance rate to oseltamivir is generally low (1%–3%), and resistance to zanamivir is rare (<1%) [6,7,8] The high resistance barrier of zanamivir is likely explainable by its higher conformational similarity to the natural substrate sialic acid thus affinity to the active site of viral neuraminidase (compared with peramivir and oseltamivir), and the extremely high topical concentration delivered through inhalation [6]. The less extensive use of this agent is another possible reason. Notably, propensity of emergence of oseltamivir resistance seems to differ between virus subtypes with different neuraminidase structures. It has been reported that resistant strains emerge more commonly among oseltamivir-treated children infected with the pre-pandemic A/H1N1 than A/H3N2 viruses (2005–2007; 3/11 (27%) *vs.* 1/34 (3%)) using a tiered weight-based regimen [9]. The resistant A/H1N1 viruses were found to harbor the H275Y mutation (single neuraminidase amino acid H275Y substitution, N1 numbering). During the 2007–2008 influenza season, an oseltamivir-resistant H1N1 (A/Brisbane/59/2007-like) virus, characterized by the H275Y mutation, emerged first in Europe among persons without antiviral exposure; it quickly spread to North America and then the Asian-Pacific countries within months, and became the predominant circulating strain globally [10,11,12,13]. In contrast to earlier H275Y mutants with reduced “viral fitness”, this H1N1 strain was readily transmissible, causing severe outbreaks and high mortality similar to the drug-susceptible viruses, owing to the presences of several permissive, “compensatory” mutations (e.g., R194G, R222Q, V234M, and D344N, N1 numbering) [6,12,14,15,16,17]. *In vitro* susceptibility testing showed high-level oseltamivir resistance (50% maximal inhibitory concentration (IC_50_) increase by several hundred-folds) as the mutation affected drug binding to the active site; clinically, lack of efficacy was observed [18,19]. Zanamivir binding was unaffected, as well as the M2-inhibitors [12]. As such, zanamivir or an adamantine-containing regimen had been recommended for empirical therapy during the period; available evidence suggested that use of a susceptible agent may reduce adverse outcomes [18]. Use of inhalational zanamivir in patients hospitalized with severe influenza can be challenging [1]. This virus was later replaced by A/H1N1_pdm09_ in 2009; however, the event highlights the risk of a transmissible drug-resistant virus to cause a pandemic, if given the suitable backbone to maintain replicative “fitness” and virulence [14,17].

Although the A/H3N2 viruses are generally susceptible to NAI, secondary resistance (characterized by E119V or R292K substitutions, N2 numbering) do occur [6]. The two most well-reported “at-risk” groups are young children and the immunocompromised, as explainable by their high virus burden and prolonged duration of viral replication. In an earlier report, resistant strains were identified in 18% of young children treated with oseltamivir, although under-dosing might have contributed to this high incidence [20]. Later reports in this patient group showed a lower rate (2%–8%) [8,21]. There are numerous reports documenting resistant A/H3N2 strains emerging during prolonged courses of oseltamivir in immunosuppressed individuals, leading to therapeutic failure; in some cases a combination of mutations occurs, resulting in reduced susceptibility to peramivir and even zanamivir [6,22,23,24,25]. Since the early 2000s, all circulating A/H3N2 strains globally have become resistant to adamantanes as a result of a S31N amino acid substitution in the M2 protein (ion channel pore) [12].

Influenza B is noted to respond slower to oseltamivir, in terms of viral clearance and clinical resolution, than influenza A (in both children and adults); treatment with zanamivir show better responses [26,27,28]. These observations are consistent with data on oseltamivir IC_50_ of clinical influenza B virus isolates which show values 10–100 folds higher than those of influenza A (in a recent study, 1.4–2.4 ng/mL *vs.* 0.1–0.2 ng/mL, respectively), but it remained low with zanamivir [6,8]. In a recent clinical trial among hospitalized adults, high-dose oseltamivir treatment (150 mg bid) was shown to improve viral clearance in influenza B [29]; no advantage was observed for influenza. A viruses, as predicted by their lower IC_50_ in relation to the attainable oseltamivir levels. Notably, data from peramivir clinical trials showed a superior virologic response than oseltamivir in influenza B in adults [30]. Recently, community clusters of influenza B infections with reduced susceptibility to oseltamivir (e.g., I221V/T, influenza B numbering) have been reported, in the absence of prior drug exposure, raising again the concern of a “fit”, transmissible resistant virus [6,12,31,32,33]. New data suggest that resistant-associated mutations may affect susceptibility to a different extent among the two vaccine-covered B-lineages (B/Victoria, B/Yamagata) [34].

## 3. Pandemic Influenza Virus, A/H1N1pdm09

The A/H1N1pdm09 virus which caused a pandemic in 2009, has continued to circulate; on-going surveillance data indicate that the incidence of NAI resistance has remained low (<3%) [6,7,8,12,35,36]. Early in the pandemic, oseltamivir-resistant, H275Y-harbouring mutants typically emerge during drug exposure among the “at-risk” groups, e.g., young children 1–5 years, hematological oncology, and transplant patients (overall, immunocompromised patients constitute >27% of resistant cases) [37,38]. Although resistance is usually observed after 11–23 days of oseltamivir treatment in the immunocompromised, early *de novo* occurrence as early as two days has been reported [39]. In some cases, a mix of wild-type and H275Y strains in the original virus population was detected, and the latter overgrow under drug selection pressure [40]. These resistant strains are capable of transmission, and have caused nosocomial outbreaks involving immunocompromised patients [6,41,42]. Besides, the use of “half dose” oseltamivir (75 mg daily) for chemoprophylaxis and outbreak control during the pandemic (e.g., households, school camps), had been associated with emergence of resistance, likely attributable to the sub-therapeutic drug levels achieved in an infected individual [6,43,44]. Subsequently, it is recommended that the strategy of early detection and treatment with a full dose regimen should be used, if considered necessary to control an outbreak [45,46]. Whether use of inhalational zanamivir as prophylaxis has an advantage because of its higher resistance barrier deserves investigation [41].

Although the overall incidence remains low, more recent data show a rising proportion of resistant cases involving non-immunocompromised community dwellers without prior exposure to oseltamivir (e.g., 11%–74% in US; >14% globally), which suggest limited community transmission of resistant viruses [6,38,47]. H275Y mutants caused community outbreaks in Australia and regions in Asia, raising great concern for a more widespread, sustained transmission [12,48,49,50]. It is suggested that several permissive NA mutations (e.g., V241I, N369K, N1 numbering) in the more recent A/H1N1pdm09 isolates might have contributed to the emergence of H275Y mutants due to restoration in replicative and transmission “fitness” and virulence [12,17,38,51,52]. Further monitoring of their circulation is essential.

Besides exhibiting high-level oseltamivir resistance (about 200–1200 times of wild-type virus), the A/H1N1_pdm09_ H275Y mutant shows cross-resistance to the newly approved peramivir, but not zanamivir; and it is not susceptible to M2-inhibitors [5,12,38]. Due to disruption in active site binding, peramivir IC_50_ increases by 100–400 times; whether intravenous administration of high-dose peramivir may overcome the reduced susceptibility, as suggested in an animal model, are controversial [51,52,53]. However since H275Y emergence has been reported during peramivir therapy [54,55], its use in a known or suspected H275Y case is not advisable. In immunocompromised patients with prolonged viral shedding, additional, sequential mutations may occur (e.g., H275Y and I223R, N1 numbering), resulting in clinical resistance to multiple NAIs (oseltamivir, peramivir, and zanamivir) and uncontrolled infection [6,38,42,56,57,58].

## 4. Avian Influenza Viruses, A/H5N1, A/H7N9

Data on human A/H5N1 infections in endemic areas have shown infrequent resistance to oseltamivir and zanamivir [6,59,60]. In the largest cohort study published to date (*n* > 400), oseltamivir treatment significantly improved patient survival if started within two days, and remained beneficial (albeit smaller) until 6–8 days after onset [61,62], which was explainable by the prolonged viral replication in such cases. Susceptibility to adamantanes is variable across regions, depending on the lineage (“clade”) of virus in circulation (e.g., clade 1.1, clade 2.1.3; some clade 2 isolates also have reduced susceptibility to oseltamivir) [6,12,60]. In susceptible cases, combination therapy with an NAI has been proposed, since synergistic actions have been observed in animal studies (discussed below) [1,4]. Secondary resistance (typically H275Y, N1 numbering) may emerge during the course of oseltamivir therapy, resulting in fatality [60,63]. As such, monitoring response and viral load changes are important during management of avian influenza. In 2013, a novel A/H7N9 virus emerged in China, which has become endemic. Human infections predominantly occur in relation to exposure to poultry (e.g., wet market), although limited human-to-human transmission has been reported [64]. *In vitro* testing indicates its susceptibility to oseltamivir, peramivir, and zanamivir, but resistance to adamantanes (S31N mutation, M2 numbering) [12]; and use of NAI treatment has been associated with clinical improvements [64]. Expectedly, secondary resistance can occur during the course of therapy in severe diseases (e.g., R294K, N9 numbering), leading to viral load rebound and clinical progression [65]. Notably, detection of phenotypic resistance can be difficulty, as there could be a mix of resistance and wild-type viruses [66]. The R294K mutant shows high-level resistance to oseltamivir and peramivir, and moderately reduced susceptibility to zanamivir and laninamivir [66,67]; additionally, replication and transmission “fitness” does not seem to be compromised (unlike the R292K mutant in A/H3N2) [12,67,68,69]. Very limited data exists on its incidence; close surveillance using appropriate laboratory methods is necessary.

## 5. Detection and Clinical Management

The importance of continuous laboratory surveillance, and detailed techniques and methodological challenges for the detection of influenza virus resistance has been reviewed elsewhere [38,51,70,71]. In the clinical setting, recognizing and diagnosing resistance can be challenging (Table 3). Assessment for risk factors (e.g., immunocompromised patients, young children, novel/avian influenza, history of “half-dose” NAI prophylaxis), monitoring for clinical responses and serial viral load changes in the hospitalized patients (e.g., viral rebound), and collection of appropriate clinical specimens for testing are important steps [38,51,71]. Since viral replication is much more prolonged in the lower respiratory tract in cases of influenza pneumonia, obtaining tracheal aspirates or BAL for virus detection (instead of nasopharyngeal samples), particular in patients intubated for mechanical ventilation, should be considered to avoid false-negative results [1,72]. Analysis of samples collected prior to initiation of antiviral treatment (e.g., that used for diagnosis) in addition to subsequent serial samples will determine whether the resistant strain (exists in whole or as a subpopulation) has caused the initial infection, or emerged *de novo*. Briefly, there are phenotypic and genotypic assays for resistance testing, which are complementary [51,70]. Phenotypic assays provide results on the degree of susceptibility (e.g., IC_50_ and IC_90_ values) to an individual antiviral agent, but definitions for “resistance”, *i.e.*, values predictive of clinical failure, are evolving (e.g., for influenza A, reduced inhibition as 10–100-fold increase in IC_50_; highly reduced inhibition as >100-fold increase), and variations in laboratory methods have added to the complexity for interpretation [6,38,46,51,73]. Detection of resistance caused by previously unknown or uncommon mutations is possible. The original isolate should be tested, as a subpopulation of resistant mutant may be lost during cell culture propagation; even so, a minority subpopulation may go undetected with phenotypic assays [38]. Genotypic assays may provide a rapid and more accessible means for detection of resistance, if the mutation is already known. It is particularly useful if the target mutation has been shown to correlate with reduced susceptibility in phenotypic assays and clinical resistance (e.g., H275Y); the rapid results may guide clinical management [2,18,74]. Newer techniques such as pyrosequencing may provide information on the relative proportions of wild-type and multiple resistant mutations important for the study of virus evolution in patients, and can be applied directly to clinical samples [51,56,75]. The latest next-generation sequencing (NGS, also known as massively parallel sequencing) has the advantage of unprecedented sequencing depth, which will allow simultaneous detection of influenza virus quasispecies harboring resistance mutations at low frequencies (<1%); its clinical applications are promising [76,77]. It should be emphasized that once resistance is suspected or confirmed, appropriate isolation precautions should be implemented to prevent nosocomial transmission [38].

**Table 3:** Detecting influenza antiviral resistance in the clinical setting and proposed management strategies.

Key Elements
**1. Assessing for risk factors:** ■ immunocompromised patients, young children, novel/avian influenza virus (due to high virus burden and prolonged duration of viral replication) ■ hospitalized patients with severe infections ■ use of suboptimal dosage of antivirals (e.g., half-dose oseltamivir prophylaxis) ■ exposure to known resistant cases (e.g., in an outbreak)
**2. High-index of suspicion:** ■ slow clinical response, or relapse of symptoms ■ slow virologic response (e.g., lack of viral load decline as evidenced by quantitative PCR; repeated culture positivity), or viral rebound during treatment
**3. Testing for resistance:** ■ serial Upper Respiratory Tract (URT) samples (e.g., nasal/throat flocked swabs, nasopharyngeal aspirates), and Lower Respiratory Tract (LRT) samples (e.g., endotracheal aspirates, bronchoalveolar lavage) if available ■ initial samples before (or early into) antiviral treatment, as well as subsequent samples should be tested ■ the original isolate from clinical samples (instead of those from propagated cultures) is preferred ■ phenotypic and genotypic assays serve complementary functions; rapid detection of a known resistance-associated mutation (e.g., H275Y) may assist management
**4. Infection control:** ■ appropriate isolation precautions should be implemented whenever antiviral resistance is suspected/confirmed to prevent nosocomial transmission.
**5. Treatment:** ■ there is no established therapy for neuraminidase inhibitor resistant influenza infections ■ intravenous zanamivir (available through a compassionate program) may be considered for the H275Y mutants ■ investigational therapies, including combination regimens and novel agents (e.g., favipiravir, nitazoxanide) have shown promising results

**Figure 1:**
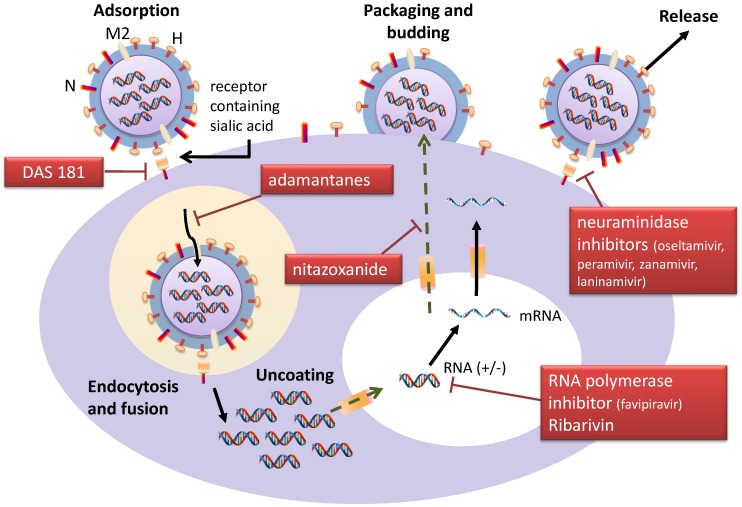
Molecular targets and potential antiviral treatments against influenza virus infection. The above diagram shows the life cycle of influenza virus and the proposed action of each class of antiviral. After attachment to the host cell receptor containing sialic acid, the virus particle undergoes the processes of fusion, endocytosis, and uncoating, and subsequently replication by the RNA polymerase. Surface protein-coated envelope then forms around the genome to produce a complete virion, which can then be released to infect other cells. DAS 181, a sialidase fusion protein, acts on the first step of virus invasion by cleaving the sialic acid linkages on human epithelial cells. Adamantanes are M2 channel blockers which inhibit proton entry through the channel into the virion, thus preventing its disintegration. Favipiravir is a pyrazinecarboxamide derivative which inhibits the viral RNA-dependent RNA polymerase. Ribavirin’s antiviral actions are multiple, though it mainly interferes with RNA synthesis. Nitazoxanide may block haemagglutinin maturation (and act as an interferon-inducer). Neuraminidase inhibitors, by attaching to the viral neuraminidase, block the release of virus from host cells, thus halting the progression of infection. A combination of agents from different drug classes may produce synergistic effects (see text).

Management of drug-resistant influenza viruses involves the use of newer/novel agents and combination treatments, which are undergoing active research (Figure 1) [4,6,38]. In cases of oseltamivir-resistant influenza infection caused by the H275Y mutation, there is cross-resistance to peramivir; zanamivir and laninamivir are unaffected and remain susceptible [51]. As the inhalational route of delivery is considered infeasible in those with severe pneumonia and critically illness [1], the investigational intravenous zanamivir has been used for viral suppression and “rescue therapy” in such cases with suspected/confirmed resistance [58,76,78,79]. Available Phase II clinical trial data on susceptible infections showed reduction in viral load and absence of drug-related trend in safety parameters [80,81]; results from a Phase III clinical trial comparing it with oral oseltamivir in hospitalized patients are pending (ClinicalTrials.gov NCT01231620). Laninamivir, an inhalational NAI given as a single dose therapy, has been shown to be non-inferior to a five-day course of oral oseltamivir in ambulatory patients [82]. Intravenous laninamivir is currently unavailable, although such treatment has been shown to offer protection against lethal influenza challenge in a mouse model [83]. Notably, combination of NAIs may result in competitive antagonism and reduced clinical efficacy, as suggested in two trials comparing the zanamivir/oseltamivir combination and oseltamivir monotherapy [4,84]. Impact of sequential therapy (e.g., oseltamivir to zanamivir; oseltamivir to peramivir) is unclear [4]. However, *in vitro* and animal data have suggested that combining antivirals from different classes (oseltamivir, amantadine, ribavirin) may be synergistic in multiple virus strains (including A/H1N1pdm09, A/H3N2, and A/H5N1), resulting in greater viral load reduction and improved survival; risk of emergence of resistance is also lower [4,84]. Interestingly, antiviral activity is observed with the “triple” combination despite oseltamivir and/or amantadine resistance at baseline [4,85]. Pharmacokinetic study shows no significant interaction between these agents [86]. In a retrospective study on critically-ill A/H1N1pdm09 influenza patients, those who received the “triple” combination (*n* = 24) showed an insignificant trend towards lower mortality than oseltamivir monotherapy (*n* = 103) (at 14 days, 17% *vs.* 35%, *p* = 0.08; at 90 days, 46% *vs.* 59%, *p* = 0.23), and little additional toxicity [87]. A randomized, controlled trial on this combination among high-risk ambulatory patients is currently under way (ClinicalTrials.gov NCT01227967).

Favipiravir (formerly known as T-705) is an oral pyrazinecarboxamide derivative that inhibits viral RNA-dependent RNA polymerase of influenza A, B, and C viruses (and many other non-influenza viruses) [88]. *In vitro* data suggest that it is active against viruses susceptible/resistant to NAI and adamantane; and synergism has been shown in combination with oseltamivir or peramivir in animal models of A/H1N1_pdm09_ and A/H5N1 influenza [4,88,89,90,91,92,93]. Phase III studies in Japan have shown antiviral effects similar to oseltamivir in uncomplicated influenza; recently, it has been approved in the country for the treatment of “novel or re-emerging influenza infections to which NAI or other antiviral agents could be ineffective” [88]. Outside Japan, a large multicenter Phase III study has just been completed, pending results (ClinicalTrials.gov NCT02008344). DAS181 is a sialidase fusion protein that acts by cleaving the Neu5Ac α(2,3)- and Neu5Ac α(2,6)-Gal linkages of sialic acid on epithelial cells, thus preventing host recognition and invasion by influenza viruses (and parainfluenza viruses). A double-blind, placebo-controlled phase II trial of inhalational DAS181 showed significant reduction in viral load in uncomplicated influenza, and was well tolerated [94]. Efficacy against drug-resistant A/H7N9 influenza has been demonstrated in a mouse model [95]. Successful therapy with this agent against severe parainfluenza pneumonia in transplant patients has been reported [96]. Oral nitazoxanide is an available, approved antiparasitic agent (e.g., against cryptosporidium, giardia) with established safety profiles. Recently, it has been shown (together with its active metabolite tizoxanide) to possess anti-influenza activity by blocking haemagglutinin maturation/trafficking, and acting as an interferon-inducer [97]. A Phase II 2b/III, randomized, placebo-controlled trial on uncomplicated influenza showed significant viral suppression and reduction in illness duration by about one day [98]. *In vitro* testing suggests possible synergy when combined with NAI [99]. A large, multicenter, Phase 3 randomized-controlled trial comparing nitazoxanide, oseltamivir, and their combination in uncomplicated influenza is currently underway (NCT01610245). Other novel agents in early stages of development (e.g., oral VX-787), and antibody-based therapies have been reviewed elsewhere [4,100].

## 6. Conclusions

In conclusion, antiviral resistance in seasonal, pandemic, and human avian influenza infections is a significant clinical and public health issue. Evidence indicates that drug-resistant viruses can retain replication and transmission fitness, thus posing global health threats. Resistance to NAIs in particular has important implications, as this class of agent is most widely used for treatment and outbreak control, and stockpiled for pandemic preparedness. Continuous laboratory surveillance efforts must be emphasized. Detection and management of drug-resistant influenza virus infections can be challenging, requiring assessment of risk factors and application of appropriate laboratory methods (Table 3). Novel and combination regimens are currently under active investigation to treat infections caused by drug-resistant viruses, and to minimize risk of resistance emergence during therapy (Figure 1).
